# Periodontal referral patterns in Norway: 2003 versus 2018

**DOI:** 10.1002/cre2.491

**Published:** 2021-09-21

**Authors:** Kristian H. Lind, Dagmar F. Bunæs, Stein Atle Lie, Knut N. Leknes

**Affiliations:** ^1^ Faculty of Medicine, Department of Clinical Dentistry University of Bergen Bergen Norway

**Keywords:** consultation, periodontitis, referral

## Abstract

**Objectives:**

Changes in periodontal referral patterns over time have been reported from the United States and Australia. To date, comparable studies have not been published from Europe. The objectives of the present study were to examine changes in periodontal referral patterns in Norway in 2003 versus 2018 and to compare these with trends observed in the United States and Australia using universal criteria for grading of periodontal severity.

**Materials and methods:**

A retrospective analysis of 369 charts from four Norwegian periodontics clinics was completed. Data on year of referral, gender, age, tobacco smoking, periodontal status and missing teeth at initial examination, teeth planned for extraction, and periodontal case type were collected using a survey format; case type I, II, III, and IV representing increasing severity of periodontitis, case type V representing referral for other periodontal conditions (peri‐implantitis, refractory periodontitis, etc.). Chi‐square, *t*‐tests, and negative binomial regression were used for the statistical analysis.

**Results:**

Compared with 2003, the 2018 data showed an increase in mean age at referral (*p* < 0.05), overall distribution of case type III and V (*p* = 0.047), and number of missing teeth (*p* = 0.001). Further, a decrease in prevalence of smokers (*p* < 0.05), but no change in number of teeth planned for extraction (*p* = 0.104), were observed.

**Conclusions:**

During a period of 15 years, changes in periodontal referral patterns in Norway are similar to those in the United States and Australia. The adoption of a guideline‐based referral practice might be beneficial for both the dental profession and patients.

## 1 INTRODUCTION

The outcome of periodontal therapy is highly dependent on early diagnosis, an appropriate treatment plan, and optimal primary and supportive periodontal therapy. At a tooth and site level, furcation involvements, tooth mobility, and infrabony defects are commonly recognized factors promulgating tooth loss (McGowan et al., 2017). At a patient level, disease modifiers including smoking and diabetes mellitus likely impair treatment outcome (Tonetti et al., 2018). Diagnosed early, periodontal disease can be successfully managed with limited morbidity using mostly noninvasive means, individualized patient supervision, and behavior modification (Axelsson et al., 2004; Saito et al., 2011; Wylam et al., 1993).

Over the last 30 years increased understanding of the pathogenesis of periodontal disease, the potential relationship between periodontal disease and systemic diseases, increased knowledge and education and a number of novel treatment modalities have contributed to present public and professional awareness and focus on periodontal disease and periodontal care needs. This has provided important reasons to investigate whether these changes have impacted periodontal referral patterns. General practitioners wish to pursue training and education to provide their patients with a broader range of “in‐house” services, including nonsurgical periodontal treatment, dental implant placement, and soft tissue and bone grafting procedures (Zemanovich et al., 2006). In addition, improved socioeconomic status in Western Europe may have affected patients' willingness to undergo periodontal treatment (Wamala et al., 2006).

General practitioners are well trained in diagnosing periodontitis and the majority of moderate to severe cases are referred to periodontal specialists. Recent reports suggest that nonclinical factors related to patients, general practitioners, practice demographics, feedback from patients, fees, and working relationships with specialty care clinics influence referral patterns (Kraatz et al., 2017, 2019). A Virginia‐based survey indicates that periodontitis receive the least referrals from general dentistry providers with less than 5 years professional experience (White et al., 2019). This might be a result of improved periodontal education over the years, but also related to the increasing debt burden faced by younger practitioners. There may also be a tendency to over‐establishment of general dentistry providers, especially in urban areas resulting in fewer patient per dentists and reduced income. Thus, universal guidelines for referral of periodontal cases based on valid clinical criteria can be warranted.

Two retrospective studies compared charts of patients referred for periodontal specialty care in the United States (Cobb et al., 2003) and Australia (Brown et al., 2017) over 20 and 15 years, respectively. Both studies concluded that comparatively fewer severe cases were referred in the first cohort compared with patients in the second. This trend was clear even though the prevalence of smoking was reduced during the same time periods. Both reports concluded that it is the responsibility of general dentistry providers to ensure that patient periodontal treatment needs are identified early, and if indicated, referred for specialty care.

The British Society of Periodontology has presented guidelines for a periodontal referral policy (Dowell & Chapple, 2002). Efforts have also been made by the American Dental Association and the American Academy of Periodontology to develop a universal screening tool for periodontal treatment needs (Charles & Charles, 1994). Comparable guidelines have not been presented in Norway or reported from the European Union. The adoption of a guideline‐based referral practice might be of significant benefit to the practice of general dentistry and patients alike. The objective of this retrospective study was to assess periodontal referral patterns in Norway in 2003 versus 2018 by comparing charts of patients referred for periodontal specialty care. A second objective was to compare referral trends in four specialist clinics in Norway with referral trends in three specialist clinics in the United States and Australia using the same criteria for grading periodontal disease severity.

## 2 MATERIAL AND METHODS

The present study closely adopted the study design presented by Cobb and colleagues reviewing periodontal referral patterns in the United States (Cobb et al., 2003). The study was approved by the Norwegian Regional Ethics Committee (Reference: 2018/2401/REK Vest).

**Figure 1:**
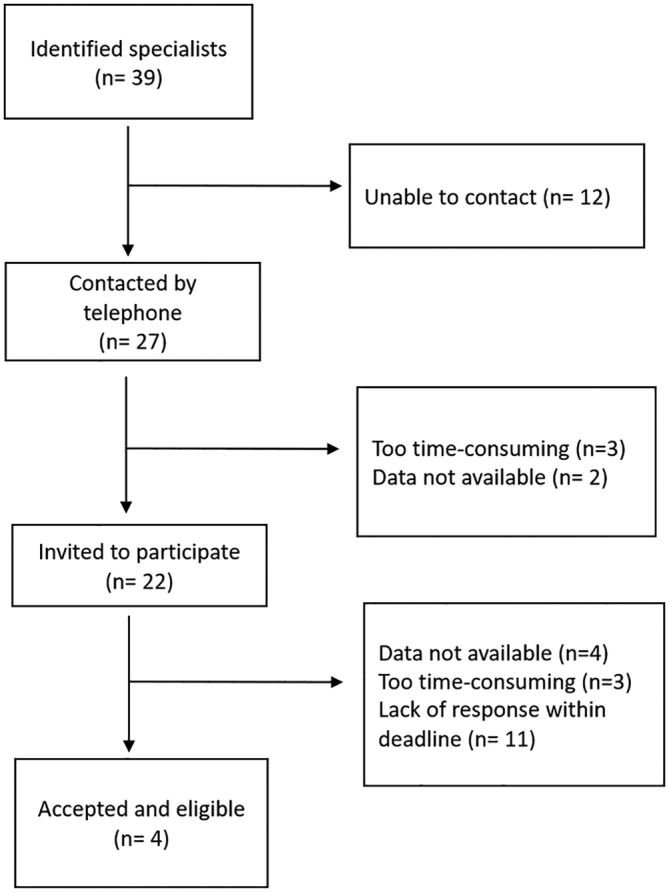
Study flowchart

The inclusion of private periodontal practices was based on the following criteria: (1) Authorized as a periodontal specialist no later than 2001, (2) Utilizing digital charting system in 2003, and (3) Willingness of the specialist to collect the data and record them on to a prefabricated, validated survey form. Written consent from patients was waived due to the anonymous nature of the survey form. An internet search, cross checked with Norwegian health worker register office (HPR), identified 39 potential specialist clinics (Figure 1). All specialists were approached by telephone. Of these, 27 were successfully contacted, 12 were not possible to reach. Five declined to participate; three because it was too time‐consuming and two due to lacking data. The 22 remaining agreed to receive an email invitation to participate in the study. Four of these did not have available data, three declined to participate due to lack of time, and 11 failed to respond within the deadline. Four periodontists thus agreed to provide research data. The second objective of the study was to compare the outcomes with previous findings from the United States (Cobb et al., 2003) and Australia (Brown et al., 2017). In order to assure a valid comparison, the present study replicated the design of the above publications, including the same variables and classification system for periodontal severity (Oliver & Heuer, 1995). The definitions of periodontal case type are shown in Table 1.

**Table 1:** Criteria for grading periodontal severity

Case type	Features
I	Gingivitis, no clinical or radiographic evidence of attachment loss
II	CP of slight severity, PD: 3–4 mm; radiographic evidence of resorption of interproximal crestal lamina dura
III	CP of moderate severity; PD: 4–6 mm; radiographic evidence of alveolar bone resorption; Class I or II tooth mobility and Class I or II furcation involvement
IV	CP of advanced severity; PD = 4–≥7 mm; Class I, II, or III tooth mobility and Class I, II, or III furcation involvement
V	Various periodontal conditions not designated above, including peri‐implantitis, aggressive periodontitis, necrotizing periodontal disease, and refractory periodontitis

### 2.1 Data collection

Four private periodontal specialty care clinics were found eligible and accepted to be included for a retrospective chart analysis. Two clinics were located in Eastern and two in Western Norway. The collection of data was undertaken within each clinic from August 2019 through February 2020. Generally, charted data from the first 50 patients referred to each clinic for periodontal specialty care in 2003 and 2018 were retrieved and tabulated. If the clinic received less than 50 referrals either in 2003 or 2018, data from all referred patients were retrieved. To ensure consistent and standardized data collection, the designed survey form was used. Unclear guidelines were discussed by telephone. The following data were collected from each patient chart: (1) year of referral, (2) gender, (3) age, (4) missing teeth at initial examination (not including third molars), (5) teeth planned for extraction due to hopeless prognosis prior to treatment, and (6) periodontal case type. The specialist filled out the survey form based on digital charting and referral notes and then returned it by mail to the University of Bergen.

### 2.2 Statistical analysis

Data were recorded and entered into MS‐Excel (Microsoft, Redmond, WA), proofed for errors, and subsequently imported into Stata, version 16 (StataCorp, College Station, TX). For comparison of categorical variables, Chi‐square tests were used, and *t*‐tests were used for comparison of continuous variables between groups. For extended and adjusted analysis of number of missing teeth and number of teeth planned for extraction, negative binomial regression was used. In these regression analyses, period (year), age, gender, and location were included. *P*‐values less than 0.05 were considered statistically significant. Patients referred to four representative clinics were the statistical unit in all analyses.

## 3 RESULTS

Patient demographics for 2003 and 2018 are presented in Table 2. Three hundred sixty‐nine surveys meeting qualifying criteria were received; 180 from 2003 (51.7% female) and 189 from 2018 (54.5% female). Patient mean age was 52.3 (±13.6) years, range 20–84 years. Mean age at referral was significantly higher in 2018 than in 2003 (*p* < 0.05). The prevalence of smokers was reduced from 78 (44%) in 2003 to 62 (33%) in 2018 (*p* = 0.032). The decline was caused by a significant reduction in female smokers (*p* = 0.006) but not male smokers (*p* = 0.826).

**Table 2:** Patient characteristics for 2003 and 2018

	2003			2018		
Location	*n* (%)	Mean age years (*SD*)	Smokers *n* (%)	*n* (%)	Mean age years (*SD*)	Smokers *n* (%)
Stavanger						
Males	23 (47)	48 (±10)	11 (48)	20 (38)	52 (±12)	8 (40)
Females	27 (53)	47 (±8)	13 (48)	33 (62)	57 (±17)*	10 (30)
Total	50	48 (±9)	24 (48)	53	55 (±15)*	18 (34)
Egersund						
Males	23 (55)	43 (±10)	4 (17)	20 (43)	54 (±15)*	3 (17)
Females	19 (45)	42 (±8)	8 (42)	27 (57)	51 (±16)*	5 (19)
Total	42	43 (±9)	12 (29)	47	52 (±15)*	8 (18)
Total West	92	45 (±9)	36 (39)	100	54 (±15)*	26 (27)
Oslo						
Males	20 (51)	54 (±12)	8 (44)	18 (46)	58 (±14)	6 (33)
Females	19 (49)	52 (±13)	11 (58)	21 (54)	52 (±17)	7 (33)
Total	39	53 (±12)	19 (51)	39	55 (±16)	13 (33)
Hønefoss						
Males	21 (43)	56 (±9)	8 (42)	28 (56)	61 (±13)	13 (46)
Females	28 (57)	48 (±10)	15 (54)	22 (44)	59 (±15)*	10 (45)
Total	49	52 (±11)	23 (48)	50	60 (±14)*	23 (46)
Total East	88	52 (±11)	42 (49)	89	58 (±15)*	36 (40)
Study total						
Males	87 (48)	50 (±11)	31 (37)	86 (46)	57 (±14)*	30 (36)
Females	93 (52)	48 (±11)	47 (51)	103 (54)	55 (±16)*	32 (31)*
Total	180	49 (±11)	78 (44)	189	56 (±15)*	62 (33)*

Abbreviation: SD, standard deviation.

^*^ *p* < 0.05.

In 2003, only three type I cases (gingivitis) were referred, whereas the corresponding number in 2018 was one (not tabulated). We found an overall statistically significant difference in the distribution of periodontal case type, with an increase in type III and V and a subsequent decrease in type II and type IV from 2003 to 2018 (*p* = 0.047). Table 3 shows the distribution of median case type and periodontal severity (%) across locations for the two cohorts. Stratified by region, the Eastern part showed the same pattern (*p* = 0.020), while the Western part exhibited no change in case type distribution (*p* = 0.589).

**Table 3:** Periodontal severity across locations for 2003 and 2018

Location	Median case type (SIR)	Type II (%)	Type III (%)	Type IV (%)	Type V (%)	*P*
Stavanger						
2003	4 (0)	2.0	4.0	94.0	0.0	0.151
2018	4 (0)	3.8	13.2	79.2	3.8	
Egersund						
2003	3 (0)	4.8	73.8	21.4	0.0	0.283
2018	3 (1)	2.3	61.4	36.4	0.0	
Total West	4 (1)	3.2	35.5	60.3	1.0	0.384
Oslo						
2003	4 (0)	13.2	5.3	81.6	0.0	0.120
2018	4 (0)	7.7	7.7	71.8	12.9	
Hønefoss						
2003	4 (0)	8.3	10.4	77.1	4.2	0.061
2018	4 (1)	14.3	26.5	51.0	8.2	
Total East	4 (1)	10.9	13.2	70.0	6.3	0.007
Study total						
2003	4 (1)	6.7	22.5	70.0	1.1	0.047
2018	4 (1)	7.0	27.0	60.0	6.0	
Total	4 (1)	6.9	24.8	64.7	3.6	

Abbreviation: SIR, semi‐interquartile range.

Categories of missing teeth and teeth planned for extraction for the two cohorts are presented in Table 4. There was an overall change in the distribution of missing teeth from 2003 to 2018 (*p* = 0.008), with an increase in categories 5–8, and ≥9 missing teeth. Stratified by region, there was no statistical changes in the distribution of missing teeth categories. The total number of missing teeth for all patients was 686 in 2018, whereas the corresponding number in 2003 was 409 (*p* = 0.001).

**Table 4:** Distribution of categories of missing teeth and planned extractions for patients across locations for 2003 and 2018

		Categories of missing teeth					Categories of teeth planned for extraction			
Location	*n*	0	1–4	5–8	≥9	*P*	0	1–4	≥5	*P*
Stavanger										
2003	50	38.0%	42.0%	14.0%	6.0%	0.735	80.0%	18.0%	2.0%	
2018	53	32.1%	39.7%	17.0%	11.2%		78.9%	13.5%	8.6%	0.241
Egersund										
2003	42	40.5%	50.0%	2.4%	7.1%	0.101	100%	0.0%	0.0%	
2018	46	30.4%	41.3%	15.3%	13.0%		95.6%	2.2%	0.0%	0.250
Total West	191	35.0%	43.0%	12.5%	9.5%	0.148	87.9%	8.9%	3.2%	0.117
Oslo										
2003	37	46.0%	45.9%	8.1%	0.0%	0.642	61.5%	38.5%	0.0%	
2018	35	42.9%	42.8%	11.4%	2.9%		71.8%	25.6%	2.6%	0.315
Hønefoss										
2003	49	14.3%	73.5%	7.4%	4.8%	0.069	69.4%	28.6%	2.0%	
2018	49	20.4%	49.0%	12.2%	18.4%		71.4%	26.6%	2.0%	0.975
Total East	170	28.8%	54.2%	10.0%	7.0%	0.053	68.8%	29.6%	1.6%	0.577
Study total										
2003	181	33.1%	52.5%	8.3%	6.1%	0.008	77.4%	21.0%	1.7%	0.104
2018	189	29.6%	41.8%	13.8%	14.8%		78.3%	16.4%	5.3%	
Total	370	31.4%	47.0%	11.1%	10.5%		77.8%	18.7%	3.5%	

In 2003, the total number of teeth planned for extraction was 91 (180 patients), while the number increased to 105 (189 patients) in 2018. The difference was nonsignificant (*p* = 0.104; not tabulated).

For missing teeth, the regression analyses for 2003 and 2018 indicated a significant effect of location (*p* = 0.001) and year (*p* < 0.001), but not gender (*p* = 0.473) and smoking (*p* = 0.082). Corresponding analyses for planned extractions, revealed a significant effect of location (*p* < 0.001) and gender (*p* = 0.002), whereas year and smoking were nonsignificant (*p* = 0.372 and *p* = 0.277, respectively).

## 4 DISCUSSION

The following trends were observed over time from 2003 to 2018 in the present study: a significant increase in mean referral age; a significant increase in the distribution of case types III and V; a significant increase in number of missing teeth; and a significant decline in prevalence of smokers. There was no significant change in distribution of teeth planned for extraction. One may speculate if these changes over time may delay the diagnosis, screening, and referral of periodontitis cases, and have caused inadequate or lack of treatment. As the distribution of case type V also increased, a higher number of teeth with questionable prognosis was most likely extracted and prosthetically replaced. Nonclinical factors as patient anxiety or fear of treatment and financial situation may further hinder or delay timely referral. The overall findings may indicate that general practitioners to a greater extent in 2018 treated uncomplicated cases and referred the most severe ones.

The present findings indicate a significant increase in the mean age of the referred patients from 49 years in 2003 to 56 in 2018. A similar trend over time was observed in USA (7‐year increase) over a 20‐year period (9) and in Australia (5‐year increase) over a 15‐year period (10). This increase may be related to a growing proportion of elderly people (Ainamo & Osterberg, 1992) who have lost fewer teeth (Hopcraft, 2015). In Australia, the proportion of +65 year olds is steadily increasing. In 2004, 13% of the Australian population were +65 years old with a projected increase to 16.8% by 2023. At the same time, the number of edentulous individuals is decreasing. Projections indicate that the proportion of edentulous individuals in Australia will decrease from 3% by 2021 to 1% at 2041 (Hopcraft, 2015). A growing, longer living dentate population, more prone to suffer from noncommunicable diseases (Gong et al., 2018) and potentially increasing the risk of developing periodontitis (Eriksson et al., 2019; Sanz et al., 2020), may partly explain the increased referral mean age trend from 2003 to 2018.

Furthermore, the role of general practitioners as the primary screening and referral source and patient factors may be of significance. General practitioners may see a financial gain in in‐house nonsurgical treatment by dental hygienist. In USA and Norway, nonsurgical periodontal treatment is part of the total insurance costs for dental treatment. This financial aspect may play a role in the referral decision for the general practitioner (Flemmig & Beikler, 2013; Kraatz et al., 2019; White et al., 2019). It has been stated that a major factor in failing to refer periodontitis cases to specialist services has been inaccessibility (Linden et al., 1999). The influence of “non‐disease factors” on the number of referrals is supported by Bennett et al. (2010), reporting that general practitioners with patients from lower socioeconomic backgrounds tend to generate less referrals. Another study, Kraatz et al. (2019) has suggested high cost, complex medical history, and unsuccessful motivation of patients as critical factors for timely referral. As such, nonclinical factors may influence the timepoint of referral, increase the probability of more advanced severity and possibly reduce the number of referrals.

A study by Linden et al. (1999) compared referral patterns in two regions of UK. A minority of the general practitioners in the study (15%) did full mouth periodontal recordings on their patients, whereas 68% used the Community Periodontal Index of Treatment Needs (CPITN) for periodontal assessment. Consequences of partial periodontal recordings might be underscoring of severity and extent and subsequent delayed referral. This issue has been discussed by Papapanou (2012) demonstrating that partial mouth recordings may underestimate diagnosis of severe periodontitis by almost five‐fold. When periodontal treatment is provided before the disease is classified as “severe,” the potential for tooth loss is significantly reduced. Delaying treatment until a patient has “severe periodontitis” increases the likelihood of tooth loss from ≈20% to ≈70% (Martin et al., 2010). This indicates that delayed referral to periodontist, may have crucial consequences for the treatment outcome.

Smoking has a negative impact on periodontal progression and prognosis (Bunaes et al., 2016; Dietrich et al., 2007). The current study disclosed a gender difference in smoking habits over time. Among male patients, a significant reduction in smoking status was not observed over 15 years, whereas the reduction in smoking females was highly significant. The trend in Europe, and especially in northern countries, is that fewer smoke (Manzoli et al., 2019). In Norway, there has been an overall reduction in daily smoking from 43% in 1973 to 13% in 2014 (Fardal et al., 2020; Gartner et al., 2016). In the current study, this trend was only observed in referred female patients. These findings are in contrast to published data from Norwegian health authorities, which demonstrate that the largest reduction in daily smoking is found in males, not females. Similar trends were observed in USA and Australia where, in general, decreased smoking was observed for both genders (Brown et al., 2017; Cobb et al., 2003). Differences were also found in regard to location. A significantly higher percentage of smokers were in 2018 referred to the two clinics in the Eastern part of Norway compared with the two clinics in the Western part. Generally speaking, a reduction in smokers with a concomitant increase in severity of periodontal disease is unexpected. However, this may be partially explained by the fact that overall severity and prevalence of periodontitis may increase with age, non‐disease factors, and underestimated diagnosis (Holde et al., 2017).

A slight decrease in patients with no missing teeth was observed from 2003 to 2018, 33.1% versus 29.6%, respectively. The opposite was seen in patients missing more than nine teeth, 6.1% versus 14.8%, respectively, indicating that referred patients in 2018 started their specialist treatment with fewer teeth than patients referred in 2003. The higher referral age in 2018 might partly explain these observations (Holde et al., 2017). However, these findings are consistent with data from USA (Cobb et al., 2003), whereas in Australia (Brown et al., 2017) more patients with no missing teeth during the time period were observed. In our study, three out of four locations reported a decrease in fully dentate patients from 2003 to 2018. The reason for this decrease is difficult to pinpoint. During the same observation period, an increase in periodontal severity was demonstrated. As it is reasonable to assume that teeth were extracted due to hopeless periodontal prognosis, the overall periodontal status is supposed to be less severe. In the Virginia‐based American study, (White et al., 2019) it was suggested that there was an increased pressure among general practitioners to perform in‐house periodontal treatment in order to keep the revenue for themselves, instead of referring to specialist. There was also a perception that general practitioners wanted to do implant surgery in‐house. In the current study, patients across all locations were missing significantly more teeth in 2018 (686 missing teeth) compared with 2003 (409 missing teeth). These findings are in line with observations from the Virginia‐based study. This may indicate that general practitioners first attempted to treat the periodontitis, and when unsuccessful, referred the cases (Darby et al., 2005).

In the United States study, there was a marked reduction in patients without missing teeth from 1980 to 2000 (Cobb et al., 2003). In the Australian study, no such trend was discerned. In contrast, an increase in patients without missing teeth between 2000 and 2015 was observed, patients retaining more teeth over their lifespan (Brown et al., 2017). The opposite finding was observed in regard to teeth planned for extraction. Our observations are aligned with the Australian study, where no significant changes were found in teeth planned for extraction after the initial examination. This could be explained by a shift in treatment options from 1980 to 2003 influencing practitioner's view on periodontal prognosis.

The present findings revealed a significant increase in distribution in case type III and V over the 15‐year span. This partly agrees with reports from USA and Australia demonstrating that patients referred in 2018 in general had a more severe periodontitis compared with cases in 2003. The fact that patients were referred with more advanced periodontitis in 2018, and at the same time were missing more teeth, raises the question whether adequate examination and treatment were implemented in a timely manner prior to referral. Acknowledging that periodontal severity has increased over time, may strongly support the need for universal guidelines for referral of periodontal patients. The establishment of evidence‐based guidelines for general practitioners on the appropriate referral timepoint would be beneficial to the periodontal specialists, the dental profession in general, and patients.

The current study is not exempt from limitations: (1) The retrospective nature of our study increases the risk of bias. (2) Due to incomplete data and the large workload associated with providing data, many potential periodontal practices could not be recruited. Therefore, we could not randomly select participating clinics. On the other hand, when patient is the unit of analysis, the inclusion of 369 referral charts has to be considered as a rather substantial number (Brown et al., 2017; Cobb et al., 2003). (3) The periodontists were asked to respond to a simple survey form. This did not include items providing complete information about the referred patient, such as exact indications for referral, tooth mobility and migration, bleeding, and so on, if the patient had previously been referred, why referral was potentially delayed, (4) or the reason(s) for extraction. (5) Because the practices were geographically situated widely apart, calibration on collection of patient data could not be performed. (6) There is no information regarding the number of general practitioners referring to each specialist, which might potentially skew the data. Moreover, some periodontal cases were most likely referred by dental hygienists. Despite these limitations, our study gives valuable insight in changing referral patterns in Norway over a 15‐year period.

In conclusion, an increase in patient' age and number of missing teeth at referral from 2003 to 2018 could pave the way for the development of Universal guidelines for appropriate referral to facilitate management of periodontal cases with greater predictability and less morbidity. The demand and the outline of periodontal referral guidelines among general dentistry providers and specialists should be explored in future surveys.

## CONFLICT OF INTEREST

The authors have stated explicitly that there are no conflicts of interest in connection with this article.

## AUTHOR CONTRIBUTIONS

All authors have made substantial contributions to conception and design of the study. Kristian H. Lind and Stein Atle Lie analyzed the data. Kristian H. Lind, Dagmar F. Bunæs, and Knut N. Leknes have been involved in data interpretation, drafting the manuscript, and revising it critically and have given final approval of publishing.

## Data Availability

The data that support the findings of this study are available on request from the corresponding author. The data are not publicly available due to privacy or ethical restrictions.

## References

[cre2491-bib-0001] Ainamo, A. , & Osterberg, T. (1992). Changing demographic and oral disease patterns and treatment needs in the Scandinavian populations of old people. International Dental Journal, 42, 311–322. 1483724

[cre2491-bib-0002] Axelsson, P. , Nystrom, B. , & Lindhe, J. (2004). The long‐term effect of a plaque control program on tooth mortality, caries and periodontal disease in adults. Results after 30 years of maintenance. Journal of Clinical Periodontology, 31(9), 749–757. 10.1111/j.1600-051X.2004.00563.x 15312097

[cre2491-bib-0003] Bennett, D. , Lee, J. H. , Richards, P. S. , & Inglehart, M. R. (2010). General dentists and periodontal referrals. Journal of the Michigan Dental Association, September, 46–51. 20945701

[cre2491-bib-0004] Brown, L. M. , Bowman, P. , O'Rourke, V. J. , Mercado, F. , Marshall, R. , & Parsons, S. (2017). Periodontal referral patterns in Australia: 2000 versus 2015. Journal of Periodontology, 88(9), 869–875. 10.1902/jop.2017.160774 28517973

[cre2491-bib-0005] Bunaes, D. F. , Lie, S. A. , Astrom, A. N. , Mustafa, K. , & Leknes, K. N. (2016). Site‐specific treatment outcome in smokers following 12 months of supportive periodontal therapy. Journal of Clinical Periodontology, 43(12), 1086–1093. 10.1111/jcpe.12619 27554463 PMC5132109

[cre2491-bib-0006] Charles, C. J. , & Charles, A. H. (1994). Periodontal screening and recording. Journal of the California Dental Association, 22, 43–46. 7523617

[cre2491-bib-0007] Cobb, C. M. , Carrara, A. , El‐Annan, E. , Youngblood, L. A. , Becker, B. E. , Becker, W. , Becker, W. , Oxford, G. E. , & Williams, K. B. (2003). Periodontal referral patterns, 1980 versus 2000: A preliminary study. Journal of Periodontology, 74(10), 1470–1474. 10.1902/jop.2003.74.10.1470 14653393

[cre2491-bib-0008] Darby, I. , Angkasa, F. , Duong, C. , Legudi, S. , Pham, K. , & Welsh, A. (2005). Factors influencing the diagnosis and treatment of periodontal disease ny dental practitionaers in Victoria. Australian Dental Journal, 50(1), 37–41. 15881304 10.1111/j.1834-7819.2005.tb00083.x

[cre2491-bib-0009] Dietrich, T. , Maserejian, N. N. , Joshipura, K. J. , Krall, E. A. , & Garcia, R. (2007). Tobacco use and incidence of tooth loss among US male health professionals. Journal of Dental Research, 86(4), 373–377. 17384035 10.1177/154405910708600414 PMC2582143

[cre2491-bib-0010] Dowell, P. , & Chapple, I. L. C. (2002). The British Society of Periodontology referral policy and parameters of care. Dental Update, 2002, 352–353. 10.12968/denu.2002.29.7.352 12369309

[cre2491-bib-0011] Eriksson, K. , Fei, G. , Lundmark, A. , Benchimol, D. , Lee, L. , Hu, Y. O. O. , Kats, A. , Saevarsdottir, S. , Catrina, A. I. , Klinge, B. , Andersson, A. F. , Klareskog, L. , Lundberg, K. , Jansson, L. , & Yucel‐Lindberg, T. (2019). Periodontal health and oral microbiota in patients with rheumatoid arthritis. Journal of Clinical Medicine, 8(5), 630. 10.3390/jcm8050630 PMC6572048 31072030

[cre2491-bib-0012] Fardal, O. , Skau, I. , Rongen, G. , Heasman, P. , & Grytten, J. (2020). Provision of treatment for periodontitis in Norway in 2013—a national profile. International Dental Journal, 70(4), 266–276. 10.1111/idj.12565 32334444 PMC9379169

[cre2491-bib-0013] Flemmig, T. F. , & Beikler, T. (2013). Economics of periodontal care: Market trends, competitive forces and incentives. Periodontology, 2000, 62. 10.1111/prd.12009 23574473

[cre2491-bib-0014] Gartner, C. E. , Lund, K. E. , Barendregt, J. J. , Mohamed Nor, N. , Hassan, H. , Vedøy, T. F. , & Kvaavik, E. (2016). Projecting the future smoking prevalence in Norway. The European Journal of Public Health, 27(1), 139–144. 10.1093/eurpub/ckw180 28177432

[cre2491-bib-0015] Gong, J. B. , Yu, X. W. , Yi, X. R. , Wang, C. H. , & Tuo, X. P. (2018). Epidemiology of chronic noncommunicable diseases and evaluation of life quality in elderly. Aging Medicine (Milton), 1(1), 64–66. 10.1002/agm2.12009 PMC6880701 31942482

[cre2491-bib-0016] Holde, G. E. , Oscarson, N. , Trovik, T. A. , Tillberg, A. , & Jönsson, B. (2017). Periodontitis prevalence and severity in adults: A cross‐sectional study in Norwegian circumpolar communities. Journal of Periodontology, 88(10), 1012–1022. 10.1902/jop.2017.170164 28671509

[cre2491-bib-0017] Hopcraft, M. S. (2015). Dental demographics and metrics of oral diseases in the ageing Australian population. Australian Dental Journal, 60, 2–13. 10.1111/adj.12279 25762037

[cre2491-bib-0018] Kraatz, J. , Hoang, H. , Ivanovski, S. , & Crocombe, L. A. (2017). Non‐clinical factors associated with referrals to periodontal specialists: A systematic review. Journal of Periodontology, 88(1), 89–99. 10.1902/jop.2016.160318 27452395

[cre2491-bib-0019] Kraatz, J. , Ivanovski, S. , Hoang, H. , Ware, R. S. , & Crocombe, L. A. (2019). Non‐clinical factors associated with referral to periodontal specialists. Journal of Periodontology, 90, 877–883. 10.1002/JPER.18-0642 30693957

[cre2491-bib-0020] Linden, G. J. , Stevenson, M. , & Burke, F. (1999). Variation in periodontal referral in 2 regions in the UK. Journal of Clinical Periodontology, 26, 590–595. 10487309 10.1034/j.1600-051x.1999.260905.x

[cre2491-bib-0022] Martin, J. A. , Page, R. C. , Loeb, C. F. , & Levi, P. A. (2010). Tooth loss in 776 treated periodontal patients. Journal of Periodontology, 81(2), 244–250. 10.1902/jop.2009.090184 20151803

[cre2491-bib-0023] McGowan, T. , McGowan, K. , & Ivanovski, S. (2017). A novel evidence‐based periodontal prognosis model. Journal of Evidence Based Dental Practice, 17(4), 350–360. 10.1016/j.jebdp.2017.05.006 29197436

[cre2491-bib-0024] Oliver, R. , & Heuer, S. (1995). Dental practice patterns. II: Treatment related to oral health status. General Dentistry, 43(2), 170–175. 7590151

[cre2491-bib-0025] Papapanou, P. N. (2012). The prevalence of periodontitis in the US. Journal of Dental Research, 91(10), 907–908. 10.1177/0022034512458692 22935674

[cre2491-bib-0021] Pesce, G. , Marcon, A. , Calciano, L. , Perret, J. L. , Abramson, M. J. , Bono, R., Bousquet, J., Fois, A. G., Janson, C., Jarvis, D., Jõgi, R., Leynaert, B., Nowak, D., Schlünssen, V., Urrutia‐Landa, I., Verlato, G., Villani, S., Zuberbier, T., Minelli, C., … Ageing Lungs in European Cohorts (ALEC) study (2019). Time and age trends in smoking cessation in Europe. PLoS One, 14(2). 10.1371/journal.pone.0211976 PMC6366773 30730998

[cre2491-bib-0026] Saito, A. , Ota, K. , Hosaka, Y. , Akamatsu, M. , Hayakawa, H. , Fukaya, C. , Ida, A. , Fujinami, K. , Sugito, H. , & Nakagawa, T. (2011). Potential impact of surgical periodontal therapy on oral health‐related quality of life in patients with periodontitis: A pilot study. Journal of Clinical Periodontology, 38(12), 1115–1121. 10.1111/j.1600-051X.2011.01796.x 22093073

[cre2491-bib-0027] Sanz, M. , Del Castillo, A. M. , Jepsen, S. , Gonzalez‐Juanatey, J. R. , D'Aiuto, F. , Bouchard, P. , Chapple, I. , Dietrich, T. , Gotsman, I. , Graziani, F. , Herrera, D. , Loos, B. , Madianos, P. , Michel, J.‐B. , Perel, P. , Pieske, B. , Shapira, L. , Shechter, M. , Tonetti, M. , … Wimmer, G. (2020). Periodontitis and cardiovascular diseases. Consensus report. Global Heart, 15(1), 1–23. 10.5334/gh.400 32489774 PMC7218770

[cre2491-bib-0028] Tonetti, M. S. , Greenwell, H. , & Kornman, K. S. (2018). Staging and grading of periodontitis: Framework and proposal of a new classification and case definition. Journal of Clinical Periodontology, 45, S149–S161. 10.1111/jcpe.12945 29926495

[cre2491-bib-0029] Wamala, S. , Merlo, J. , & Bostrøm, G. (2006). Inequity in access to dental care services explains current socioeconomic disparities in oral health: The Swedish National Surveys of public health 2004‐2005. Journal of Epidemiology and Community Health, 60(12), 1027. 17108297 10.1136/jech.2006.046896 PMC2465506

[cre2491-bib-0030] White, J. H. , Carrico, C. K. , Lanning, S. K. , Waldrop, T. C. , Sabatini, R. , Richardson, C. R. , & Golob Deeb, J. (2019). Virginia‐based periodontists' perceptions: Current and future trends of the specialty. Journal of Periodontology, 90(11), 1287–1296. 10.1002/JPER.18-0634 31257595

[cre2491-bib-0031] Wylam, J. , Mealey, B. L. , Mills, M. P. , Waldrop, T. , & Moskowicz, D. (1993). The clinical effectivness of open versus closed scaling and root planing on multi‐rooted teeth. Journal of Periodontology, 64, 1023–1028. 8295086 10.1902/jop.1993.64.11.1023

[cre2491-bib-0032] Zemanovich, M. R. , Bogacki, R. E. , Abbott, D. M. , Maynard, J. G., Jr. , & Lanning, S. K. (2006). Demographic variables affecting patient referrals from general practice dentists to periodontists. Journal of Periodontolgy, 77(3), 341–349. 10.1902/jop.2006.050125 16512747

